# The effect of mild to moderate knee osteoarthritis on gait and three-dimensional biomechanical alterations

**DOI:** 10.3389/fbioe.2025.1562936

**Published:** 2025-04-03

**Authors:** Jing Pan, Zhonghao Xie, Huifang Shen, Zhiguan Huang, Xiaohui Zhang, Bagen Liao

**Affiliations:** ^1^ Department of Sports Medicine, Guangzhou Sport University, Guangzhou, China; ^2^ School of Sports and Health, Guangzhou Sport University, Guangzhou, China

**Keywords:** knee osteoarthritis, biomechanics, three-dimensional, gait analysis, disease severity

## Abstract

**Background:**

Knee Osteoarthritis (KOA) is a prevalent chronic degenerative joint disease, particularly among the elderly, leading to pain, stiffness, and diminished functionality. The progression of KOA is often associated with significant changes in gait and biomechanics, yet detailed investigations of these changes—especially in early to moderate stages—remain limited. This study aims to conduct a comparative analysis of three-dimensional gait biomechanics in patients with mild to moderate KOA, in order to gain deeper insights into the early biomechanical changes associated with KOA.

**Methods:**

A case-control study design was employed, consisting of three groups: Kellgren-Lawrence (K-L) grade I KOA patients, K-L grade II KOA patients, and healthy controls, with 15 participants in each group. Kinetic and kinematic data were collected using two force plates and a three-dimensional motion capture system. Gait parameters, joint range of motion (ROM), angular velocities, and joint moments were analyzed, with a significance level of p < 0.05.

**Results:**

Grade II KOA patients showed prolonged gait cycles, wider step widths, and reduced swing phases on the affected side compared to grade I patients and controls. Grade I patients had reduced hip and knee ROM compared to controls. In the sagittal plane, grade II patients had more significant reductions in knee and ankle ROM. In the coronal plane, grade II patients had less hip and ankle ROM than grade I and controls. Horizontally, grade II patients had greater hip ROM but reduced knee ROM compared to grade I. Additionally, grade I patients showed smaller extension moments in the hip and knee than controls. Grade II patients had lower angular velocities and reduced extension and flexion moments in the hip and knee compared to controls.

**Conclusion:**

KOA induces significant biomechanical alterations in gait, which become more pronounced with advancing disease severity. These changes highlight the importance of early detection and tailored rehabilitation strategies to improve mobility and prevent further joint degeneration. Understanding the biomechanical profile of KOA at different stages is essential for developing personalized therapeutic approaches aimed at enhancing patient quality of life and reducing the societal burden of KOA.

## 1 Introduction

Knee Osteoarthritis (KOA) is a common chronic degenerative joint disease, primarily affecting the elderly population. It is characterized by joint pain, stiffness, and a gradual decline in function (Cruz-Montecinos et al., 2016). With the global aging population and rising obesity rates, the prevalence of KOA has continued to increase, becoming a serious public health issue. According to the 2021 Global Burden of Disease report, approximately 260 million people worldwide are affected by KOA (Murray, 2024). KOA imposes a substantial economic burden on healthcare systems and severely compromises patients’ quality of life, making effective prevention and treatment strategies critically important.

Current management of KOA predominantly involves non-surgical and surgical approaches. Non-surgical strategies include pharmacological therapies, physical exercise, weight management, health education, and self-management programs (Duong et al., 2023; Geng et al., 2023; Caiado et al., 2022). Although these methods can alleviate symptoms, long-term medication use may cause adverse effects, while the effectiveness of exercise and weight management varies widely among individuals, and adherence remains challenging (Duong et al., 2023; Geng et al., 2023). When conservative interventions fail, total knee arthroplasty is often considered; however, its high cost and potential complications make it a measure of last resort (Duong et al., 2023). Compounding these challenges, existing diagnostic modalities lack sufficient sensitivity and specificity in early-stage detection, while the absence of targeted interventions addressing pathological biomechanical alterations impedes efforts to decelerate or prevent structural disease progression.

In recent years, gait analysis has emerged as a pivotal tool for evaluating functional limitations and treatment outcomes in patients with KOA (Mills et al., 2013). Multiple studies have reported that individuals with KOA often display altered gait patterns—such as reduced joint range of motion (ROM), slower walking speeds, and higher joint loading—which not only reflect the consequences of disease progression but also actively contribute to cartilage degeneration (Debbi et al., 2015; Andriacchi and Favre, 2014). Notably, these biomechanical abnormalities extend beyond the knee, affecting the hip and ankle joints through compensatory mechanisms that further disrupt normal gait mechanics (Almeheyawi et al., 2021).

Despite growing interest in KOA-related gait changes, significant knowledge gaps remain. First, research has traditionally focused on advanced KOA, paying insufficient attention to early disease stages—even though timely detection and intervention are vital for slowing disease progression and improving long-term outcomes. Second, while some studies employ two-dimensional gait parameters, comprehensive three-dimensional analyses of joint kinematics and kinetics are comparatively rare; such analyses are critical for identifying subtle gait alterations in early-stage KOA (Kour et al., 2021; Le Rossignol et al., 2023; Tanpure et al., 2024). Finally, there is a lack of systematic investigations comparing biomechanical differences across varying KOA severities, which could inform the development of personalized rehabilitation strategies tailored to patients’ specific needs at each disease stage.

To address these gaps, this study aims to recruit healthy elderly individuals and KOA patients classified as grade I and II according to the Kellgren-Lawrence grading scale. By analyzing gait characteristics and lower limb biomechanical changes in KOA patients at different stages of injury, the study aims to uncover early pathological biomechanical features of KOA. By identifying gait and biomechanical differences among KOA patients at various severity stages, this research seeks to provide targeted rehabilitation recommendations, thereby enhancing rehabilitation efficiency and delaying disease progression.

## 2 Methods

### 2.1 Subjects and measurement protocol

This case-control study was approved by the Human Research Ethics Committee of our institution (Approval No. 2022LCLL-32). The study strictly adhered to ethical standards to ensure the safety and privacy of participants. All participants were fully informed about the study’s purpose, procedures, and potential risks, and signed an informed consent form prior to participation.

The sample size was calculated using G*Power software (version 3.1.9.7) to ensure the robustness of statistical results. The study design included three distinct groups, and statistical analysis was performed using one-way analysis of variance (One-way ANOVA). The parameters set for the calculation included an effect size of 0.7 (Landry et al., 2007), statistical power of 0.95, and a significance level of 0.05. The calculation indicated that a total of 36 participants were required, with 12 participants in each group. However, to ensure data validity and account for potential dropouts, 15 participants were recruited per group, resulting in a total sample size of 45 participants.

Participants for this study were selectively recruited from the Sports Rehabilitation Center of Guangzhou Sport University. Inclusion criteria were: (1) males and females aged between 55 and 70 years; (2) diagnosis of KOA according to the 2019 American College of Rheumatology (ACR) criteria (Kolasinski et al., 2019); (3) unilateral mild KOA with Kellgren-Lawrence (K-L) grade I or II (Li et al., 2022).

Exclusion criteria were: (1) patients with a history of knee replacement or other knee surgeries; (2) patients with severe musculoskeletal diseases (e.g., hip joint disease, spinal disease; (3) patients with cognitive disorders, language barriers, or other conditions that may affect experimental compliance and data accuracy; (4) patients with severe heart disease, lung disease, or other systemic diseases that could affect gait or prevent them from completing the gait analysis test.

### 2.2 Data collection and processing

Before data collection, participants were instructed to remove personal accessories and wear standardized black shorts and a T-shirt to minimize artifacts caused by clothing. Height and weight were recorded for normalization of kinetic data.

A Vicon Nexus (Oxford, United Kingdom) optical motion capture system with ten high-speed infrared cameras (sampling frequency: 100 Hz) was used to track lower-limb movements. Reflective markers were placed on 34 anatomical landmarks, including the anterior superior iliac spine and posterior superior iliac spine on the pelvis; a cluster of four markers on the lateral mid-thigh; the lateral and medial epicondyles of the knee; a cluster of four markers on the lateral mid-shank; the lateral and medial malleoli at the ankle; and heel, first metatarsal head, and fifth metatarsal head (Pan et al., 2023).

Joint kinetics and ground reaction forces were recorded using two embedded force plates (AMTI OR6-7, United States, 60 × 40 cm) at a sampling frequency of 1,000 Hz. These force plates provided synchronized force data to compute joint moments and loading characteristics during gait.

A static standing calibration trial was performed first to determine individual joint centers and segment orientations. Participants then walked along a 6-m walkway at a self-selected speed to ensure a natural gait. A trial was considered valid if one foot completely contacted the first force plate, while the contralateral foot fully contacted the second plate. Participants performed multiple practice trials before formal testing, and at least three valid trials were recorded per participant.

Following data collection, raw kinematic and kinetic data were preprocessed in Vicon Nexus software, where marker trajectories were filtered and interpolated to correct for noise or missing frames. The processed data were then transferred to Visual 3D software (version 2.7.10, United Kingdom) for biomechanical calculations. A low-pass Butterworth filter was applied, with cutoff frequencies of 10 Hz for kinematic signals and 50 Hz for ground reaction forces, to remove high-frequency noise while preserving signal integrity.

Gait events such as heel strike and toe-off were identified using force plate and kinematic data. A full gait cycle was defined as the time between two consecutive heel strikes of the same foot, and data were normalized to 101 time points for inter-subject comparison. Joint moments were calculated via inverse dynamics analysis and normalized to body weight. For comparative analysis, the KOA-affected limb was analyzed against the dominant limb of control participants, with dominance determined based on the preferred leg for kicking a soccer ball (Vauhnik et al., 2008).

### 2.3 Data analysis

All statistical analyses were conducted using SPSS software (version 26.0, IBM Corp., Armonk, NY, United States). Data normality was assessed using the Shapiro–Wilk test, differences between groups were evaluated using one-way analysis of variance (ANOVA) for demographic and biomechanical variables. When significant differences were found, *post hoc* Bonferroni corrections were applied to account for multiple comparisons. A significance level of p < 0.05 was used, and effect sizes (Cohen’s d) were reported to quantify the magnitude of differences.

## 3 Results

### 3.1 Demographic parameters

This study consisted of three groups with 15 participants each, totaling 45 individuals. Demographic comparisons (Table 1) revealed no significant differences across the groups.

**TABLE 1:** Demographic parameters (*x̄* ± s*, n = 45*).

Parameters	CG	Grade Ⅰ	Grade Ⅱ	Control vs. Grade I		Control vs. Grade II		Grade I vs. Grade II	
				*p*	Cohen’s d	*p*	Cohen’s d	*p*	Cohen’s d
Age (years)	62.25 ± 4.18	63.50 ± 4.50	65.33 ± 5.12	0.41	0.18	0.09	0.32	0.24	0.15
Height (cm)	163.96 ± 7.13	159.39 ± 6.43	162.79 ± 9.13	0.25	0.22	0.68	0.07	0.53	0.12
Mass (*kg*)	58.89 ± 6.84	58.52 ± 7.44	63.37 ± 5.83	0.89	0.03	0.14	0.29	0.09	0.32
BMI (*kg*/*m* ^2^)	21.88 ± 2.02	22.98 ± 2.04	23.98 ± 2.20	0.12	0.26	0.10	0.32	0.17	0.18

CG, means control group; Grade I means K-L grade Ⅰ; Grade II, means K-L grade IⅠ.

BMI, Mass (kg)/Height (m)^2^.

### 3.2 Gait parameters

As detailed in Table 2, no significant differences were observed in gait parameters between the control group and K-L grade I patients. However, grade II patients exhibited a prolonged gait cycle, increased step width, and a shortened swing phase on the affected side compared to both the control group and grade I patients.

**TABLE 2:** Walking parameters among groups (*x̄* ± *s, n = 45*).

Parameters		CG	Grade Ⅰ	Grade Ⅱ	Control vs. Grade I		Control vs. Grade II		Grade I vs. Grade II	
					*p*	Cohen’s d	*p*	Cohen’s d	*p*	Cohen’s d
Gait cycle time (s)		1.09 ± 0.07	1.10 ± 0.14	1.14 ± 0.12	0.61	0.13	<0.01	0.83	<0.01	0.81
Stride/Height		0.74 ± 0.06	0.74 ± 0.08	0.74 ± 0.09	0.87	0.04	0.68	0.11	0.77	0.08
Speed (km/h)		1.11 ± 0.11	1.10 ± 0.16	1.07 ± 0.15	0.81	0.06	0.32	0.26	0.39	0.22
Step width (m)		0.07 ± 0.03	0.08 ± 0.02	0.10 ± 0.03	0.02	0.63	<0.01	1.75	<0.01	1.31
Stance time (%)	Affected	58.91 ± 2.50	58.84 ± 3.98	59.89 ± 4.04	0.90	0.05	0.13	2.63	0.07	2.64
	Non-affected	59.44 ± 1.81	59.18 ± 6.44	58.85 ± 5.39	0.76	0.08	0.52	0.17	0.69	0.10
Swing time (%)	Affected	41.19 ± 2.51	40.62 ± 2.89	39.53 ± 3.86	0.26	0.31	<0.01	0.81	0.02	0.60
	Non-affected	40.38 ± 1.50	40.45 ± 3.43	40.65 ± 3.94	0.89	0.04	0.62	0.13	0.68	0.11
Double stand time (%)		27.91 ± 4.04	18.54 ± 1.91	18.36 ± 3.02	0.82	0.06	0.88	0.04	0.67	0.11

### 3.3 Kinematic parameters

Table 3 shows that grade I patients demonstrated reduced hip and knee ROM in both the sagittal and coronal planes compared to controls, with a particularly notable reduction in hip ROM in the horizontal plane. Interestingly, the knee ROM in the horizontal plane increased in grade I compared to controls. Grade II patients exhibited further reductions in knee and ankle ROM in the sagittal plane, alongside decreased knee ROM in the horizontal plane and reduced hip mobility in the coronal plane compared to grade I patients. Additionally, maximum angular velocity of the hip and knee joints was significantly lower in grade II patients compared to controls, reflecting impaired dynamic movement efficiency as the severity of KOA progressed.

**TABLE 3:** Kinematic parameters among groups (*x̄* ± s*, n = 45*).

Parameters	CG	Grade Ⅰ	Grade Ⅱ	Control vs. Grade I		Control vs. Grade II		Grade I vs. Grade II	
				*p*	Cohen’s d	*p*	Cohen’s d	*p*	Cohen’s d
ROM in Sagittal Plane (°)									
Hip	44.10 ± 7.66	41.55 ± 3.18	42.68 ± 4.99	0.09	0.41	0.16	0.37	0.21	0.32
Knee	65.06 ± 2.84	61.92 ± 5.40	61.07 ± 4.77	<0.01	0.95	<0.01	1.16	0.28	0.27
Ankle	59.08 ± 6.53	55.60 ± 9.00	50.85 ± 5.93	0.01	0.66	<0.01	1.49	<0.01	0.97
ROM in Coronal Plane (°)									
Hip	13.65 ± 3.17	14.10 ± 3.48	12.34 ± 3.37	0.474	0.19	0.06	0.52	0.04	0.80
Knee	8.12 ± 2.77	6.85 ± 2.28	7.45 ± 2.89	0.01	0.68	0.18	0.35	0.18	0.38
Ankle	25.23 ± 4.29	21.79 ± 7.40	19.26 ± 6.58	<0.01	0.75	<0.01	1.25	0.02	0.60
ROM in Horizontal Plane (°)									
Hip	14.44 ± 4.65	11.06 ± 3.08	12.69 ± 3.31	<0.01	1.31	0.01	0.69	0.02	0.65
Knee	10.95 ± 3.33	12.87 ± 4.03	10.84 ± 2.37	<0.01	0.82	0.87	0.04	<0.01	0.92
Ankle	7.70 ± 2.71	7.86 ± 3.17	7.45 ± 3.42	0.78	0.07	0.67	0.11	0.44	0.20
Maximum Angular Velocity in Sagittal Plane (deg/s)									
Hip	198.53 ± 26.91	191.19 ± 31.25	185.64 ± 22.34	0. 16	0.36	0.04	0.68	0.22	0.31
Knee	398.06 ± 71.49	380.56 ± 52.81	362.78 ± 61.70	0.13	0.39	<0.01	0.78	0.08	0.45
Ankle	158.57 ± 48.29	160.38 ± 53.82	164.76 ± 42.78	0.84	0.05	0.51	0.17	0.59	0.13

CG, means control group; Grade I means K-L grade Ⅰ; Grade II, means K-L grade II.

### 3.4 Kinetic parameters

**FIGURE 1:**
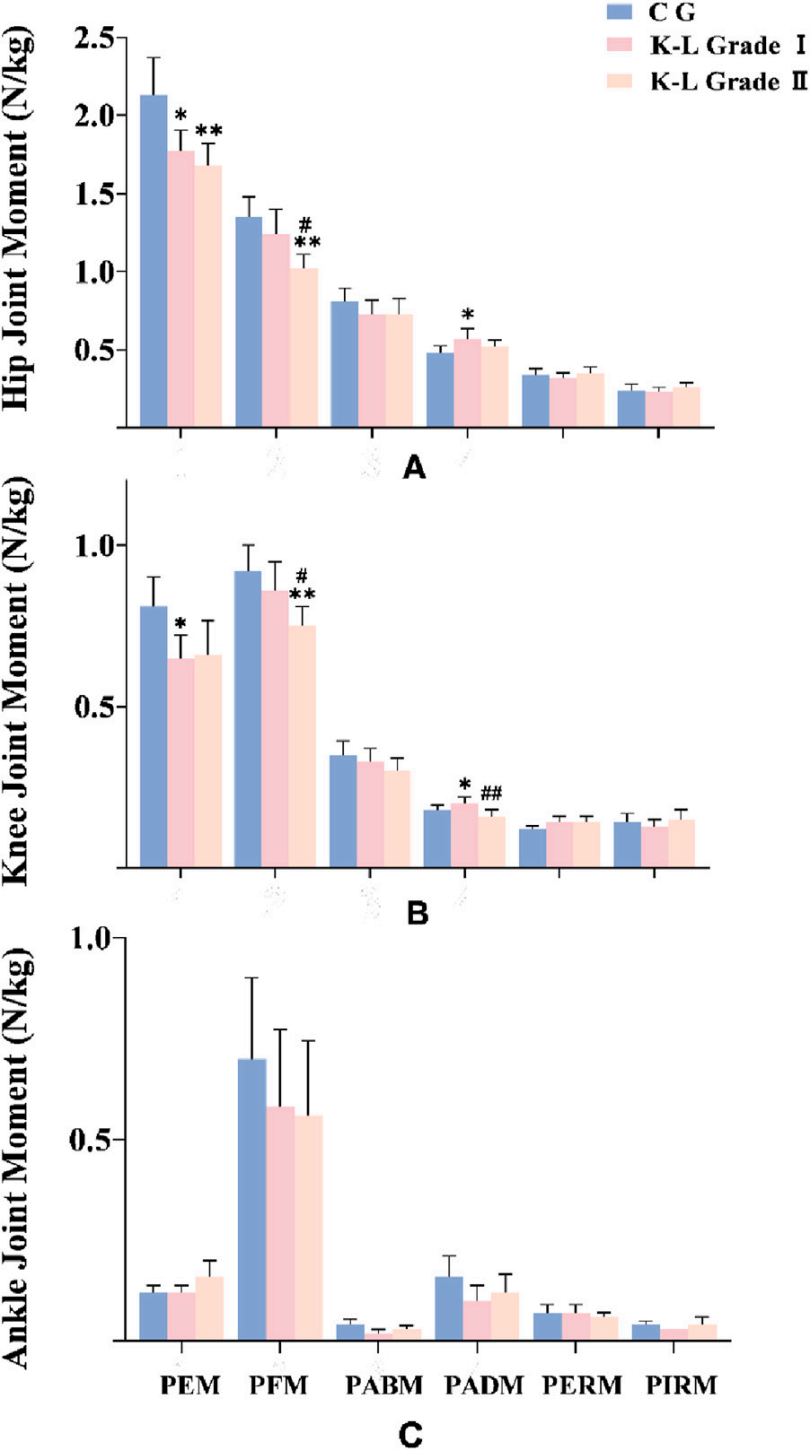
Joint peak moment among groups (N/kg). **(A)** Hip Joint peak moment among groups (N/kg). **(B)** Knee Joint peak moment among groups (N/kg). **(C)** Ankle Joint peak moment among groups (N/kg). Note: * means p < 0.05, ** means p < 0.01, compared with control group; # means p < 0.05, ## means p < 0.01, compared with K-L grade I group. PEM: peak extension moment; PFM: peak flexion moment; PABM: peak abduction moment; PADM: peak adduction moment; PERM: peak external rotation moment; PIRM: peak internal rotation moment.

As shown in Figure 1A, hip peak extension moment (PEM) was significantly lower in K-L grade I patients and further decreased in grade II patients compared to the control group. K-L grade II patients also exhibited a significantly lower hip peak flexion moment (PFM) compared to both the control group and grade I patients. Additionally, hip peak abduction moment (PABM) was significantly higher in grade I patients compared to the control group.

Figure 1B shows that knee PEM was significantly reduced in K-L grade I patients compared to the control group, while knee PFM was further decreased in grade II patients compared to both the control group and grade I. Similar to the hip joint, knee PABM was significantly higher in grade I patients compared to the control group but showed a marked reduction in grade II patients. As shown in Figure 1C, no significant differences were observed in ankle joint moments among the three groups.

## 4 Discussion

This study investigates the impact of KOA on gait and biomechanical characteristics across different stages of the disease. The findings demonstrate significant changes in gait cycle, stride length, ROM, joint moments, and angular velocity in KOA patients. These biomechanical changes not only reflect the adaptive mechanisms employed by patients in response to structural and functional impairments but also highlight the compensatory strategies underlying these gait adjustments. Importantly, the changes observed are closely related to the severity of the disease.

### 4.1 Changes in gait parameters

In this study, grade II KOA participants demonstrated significantly prolonged gait cycles and wider step widths compared to both grade I and control participants—results aligning with previous reports that KOA patients often adopt a wider base of support to enhance stability and reduce pain (Truszczyńska-Baszak et al., 2020; Boekesteijn et al., 2022). Factors such as reduced proprioception, muscle weakness, and pain avoidance behaviors may drive these adaptations (Turcot et al., 2011; Hassan et al., 2001). Although these changes can help mitigate lateral shear forces and lower fall risk, they may increase energy expenditure over time.

Interestingly, grade II participants showed a shortened swing phase on the affected side, along with a slight increase in stance phase (though not statistically significant). This adaptation appears to reduce discomfort and joint loading but can lead to atypical joint stress distribution, thereby accelerating cartilage wear (Astephen Wilson et al., 2017; Saxby et al., 2016). Future research could delve into localized joint pressures to clarify how altered stance and swing phases might promote compartment-specific degeneration.

### 4.2 Changes in kinematic parameters

This study uncovered marked limitations in both hip and knee ROM in the sagittal and coronal planes among KOA patients, exacerbated by disease severity. Similar findings have been noted in moderate-to-severe KOA cases exhibiting a “stiff knee” gait pattern, often linked to pain-induced muscle inhibition and joint damage (Astephen Wilson et al., 2017; Eitzen et al., 2015; Rutherford et al., 2017).

However, we also observed that grade I patients showed slightly increased knee ROM in the horizontal plane compared to controls. Some recent research suggests that early-stage KOA patients may compensate by subtly increasing knee motion horizontally, potentially reflecting ligamentous laxity or neuromuscular adaptations (Md et al., 2004; At et al., 2018). Nonetheless, this contrasts with the ultimate “stiff knee” gait pattern typically seen in advanced KOA, which may imply an evolution from compensatory laxity to restricted movement as the disease progresses. This abnormal accessory motion may also be a critical factor in the advancement of KOA, warranting further longitudinal tracking to clarify its significance.

Additionally, angular velocity at the hip and knee was significantly reduced in grade II KOA participants, consistent with findings of slower walking speed and an extended gait cycle in KOA patients (Saxby et al., 2016)。This pattern may represent a protective mechanism—patients consciously or subconsciously restrict joint speed to mitigate pain and prevent further damage (Ghazwan et al., 2022). Although this protective slowing can help reduce pain, it often leads to quicker fatigue and decreased gait efficiency in advanced KOA (Astephen Wilson et al., 2017; Saxby et al., 2016). Over time, a cycle of diminished joint usage can aggravate weakness and instability, reinforcing a “stiff” gait pattern (Astephen Wilson et al., 2017).

### 4.3 Changes in kinetic parameters

Regarding joint moments, Ghazwan et al. found that KOA progression is closely linked to abnormal knee loading (Ghazwan et al., 2022). Likewise, Li et al. reported that an elevated knee adduction moment constitutes a major risk factor for KOA progression (Zhang et al., 2020), a finding supported by our data. In comparison with the control group, individuals with grade I KOA exhibited a larger knee adduction moment, potentially accelerating cartilage wear.

However, KOA participants in our study demonstrated notably lower maximum extension and flexion moments in the knee and hip, and this reduction correlated with increasing disease severity. In particular, grade II KOA participants had a pronounced decrease in overall joint moments. This decline may partly stem from patients’ reduced walking speeds (Saxby et al., 2016). Previous research indicates that slower gait effectively diminishes joint moments, especially in the knee and hip (Landry et al., 2007). These observations also suggest that changes in internal joint moments may not follow a strictly linear pattern, leaving open questions about how these moment fluctuations specifically influence KOA progression. Nonetheless, it is important to note that long-term reliance on a low-moment gait strategy could alter normal knee-joint contact mechanics over time, potentially accelerating disease advancement (Andriacchi and Favre, 2014).

Interestingly, while no significant differences emerged in ankle joint moments among the groups, we observed a downward trend in the ankle’s maximum flexion moment as KOA severity increased. This finding implies that even if the ankle joint is not directly compromised by KOA, degenerative changes in the knee could initiate a chain reaction affecting the ankle’s mechanical characteristics (Almeheyawi et al., 2021; Feng et al., 2021). Consequently, KOA is not merely a knee-centric issue, but rather one that influences the entire biomechanical framework of the lower limb. From a clinical standpoint, this underscores the need to address lower-limb coordination holistically, rather than focusing solely on the knee joint.

Moreover, understanding these kinetic and kinematic alterations is crucial for guiding rehabilitation strategies. For instance, gait retraining programs can aim to optimize step width and swing time, thereby reducing abnormal frontal-plane loads and improving propulsion during the swing phase. Bracing can help realign the knee joint, mitigating excessive medial or lateral joint stresses and helping patients regain confidence in weight-bearing (Rannou et al., 2010). Assistive devices (e.g., canes, walkers) can be introduced to offload the affected knee, promote safer gait patterns, and reduce fall risk (Carbone et al., 2013). These targeted interventions, derived from biomechanical findings, can be integrated into individualized rehabilitation protocols, ensuring more efficient gait retraining while preventing further joint degeneration.

### 4.4 Limitations and future research directions

Although this study provides valuable data on gait and biomechanical changes in KOA patients, there are several limitations. First, we focused on joint-level biomechanical changes but did not analyze compensatory strategies in the trunk or contralateral limb, which are common in KOA patients. Altered trunk posture (e.g., forward trunk lean) and asymmetric loading of the contralateral limb may influence gait mechanics. Future studies should incorporate trunk and pelvic kinematics to provide a more comprehensive understanding of whole-body gait adaptations. Additionally, our statistical analysis primarily relied on discrete variables, such as peak joint angles and moments. A continuous approach, such as Statistical Parametric Mapping (SPM), could better capture variations across the entire gait cycle and should be considered in future research.

Second, this study used a marker-based motion capture system, which is susceptible to soft tissue artifacts (STA), potentially affecting measurement accuracy. Moreover, laboratory-based gait assessments may not fully reflect natural walking patterns. Future research should explore wearable sensors (IMUs) or markerless motion capture methods to improve real-world applicability. Finally, while this study identified key gait alterations in KOA patients, it did not establish diagnostic thresholds or predictive models. Given the increasing role of artificial intelligence (AI) and machine learning, future studies should evaluate how gait biomarkers can be integrated into AI-driven screening tools or longitudinal studies to monitor KOA progression and guide early interventions. Addressing these limitations will enhance the clinical relevance and translational impact of gait biomechanics research in KOA.

## 5 Conclusion

This study examined the biomechanical alterations associated with varying severities of KOA by analyzing gait, kinematic, and kinetic parameters across control, K-L grade I, and K-L grade II groups. The findings revealed that as KOA severity increased, patients exhibited significant changes in gait patterns, including a prolonged gait cycle, increased step width, and a shortened swing phase on the affected side. Kinematic analysis demonstrated reduced hip and knee range of motion in grade I patients, with further reductions in knee and ankle motion in grade II patients. Kinetic assessments showed decreased hip and knee joint moments in both patient groups, while ankle joint moments remained unchanged. These results underscore the progressive impact of KOA on lower limb biomechanics, highlighting the need for targeted interventions to preserve joint function and improve patient mobility.

## Data Availability

The raw data supporting the conclusions of this article will be made available by the authors, without undue reservation.

## Abbreviations

KOA, Knee osteoarthritis; ROM, range of motion; PEM, peak extension moment; PFM, peak flexion moment; PABM, peak abduction moment; PADM, peak adduction moment; PERM, peak external rotation moment; PIRM, peak internal rotation moment.
